# Harvey Interviewed

**Published:** 1886-12-04

**Authors:** 


### Harvey Interviewed.

By our Special Commissioner.

A cold winter's eve found me journeying in the direction of Doctors' Commons, in quest of a modest-looking habitation, whose ornamental front had apparently known better days. My errand was to see, probably for the last time, one of the greatest men which the seventeenth century had thus far produced.

I found Harvey engaged in the perusal of a medical treatise, which he closed upon my entry. What a change, I fancied, had come over the great doctor since I last saw him, when he set out with his royal master to support the Crown against the Parliament. The little, round-faced man was now in his seventy-eighth year; his black locks had turned to a whitish grey, and the lines upon his face bore silent witness to the sufferings which professional hostility and his arch-enemy, the gout, had caused him.

Ordering his servant to bring us some of his favourite coffee, he requested me to be seated, and our conversation began.

I commenced by reminding him of our former interview, when he, and I, too, were both in our prime. "Ay," rejoined Harvey, "I well remember; could I forget such a noble master as His Majesty King Charles? I threw my fortune in with his. I left London soon after I saw you, and I was with the king in that plucky fight at Edge-Hill, where poor Bertie (the Earl of Lindsey) fell. A very chill October day it was. His Majesty confided to my care Prince Charles, and his little brother James. For safety, I retired with them under a hedge, and began to read to them, but ere long the enemy sent a ball which passed in such close proximity that I hastily beat a retreat with my beloved charges."

As the conversation proceeded, Harvey grew more animated in his discourse; but when he spoke of the poor king, his sharp and penetrating eyes half filled with tears.

He reminded me how the "rebels" (the Parliament men) ransacked his lodgings in Whitehall, destroying his papers upon the dissection of frogs, toads, and other animals, a loss which neither love nor money could retrieve.

Of Oxford he told me quite a history,—of his friendship with the good master of Trinity College—Dr. Bathurst—and how they used to study the progress of egg-hatching; of his conversations with Charles Scarborough, a young medical student, who, much to the grief of Harvey, neglected his studies to take part against the Roundheads. "Prithee," said Harvey to him, "leave off thy gunning, and stay here; I will bring thee into practice."

I directed my conversation to his great discovery—the circulation of the blood—a discovery which cost him a quarter of a century's study. It was the doctrines which his teacher, Fabricius ab Aquapendente of Padua sought to teach him, which made Harvey first turn his serious attention to this great subject. He spoke, but without bitterness, of the animosity which for years was shown him by brother doctors—men who should have been the first to hail with delight such a mighty revelation to medical science.

"But," said Harvey, "I can forgive them all: I have lived to see the truth of that which I have demonstrated accepted by the world."

Still, among the many disbelievers, who would fain argue that he was either a hare-brained fellow or an arrant rogue, there were some who recognised the truth of his doctrines. One thus addresses the "incomparable doctor" on his work, *Exercitatio Anatomica de Motu Cordis et Sanguinis*:—

"There didst thou trace the blood, and first behold
What dreams mistaken sages coined of old.
For till thy Pegasus the fountain brake,
The crimson blood was but a crimson lake
Which first from thee did tyde and motion gaine,
And veins become its channel, not its chaine.
With Drake and Ca'ndish hence thy bays are curl'd,
Fam'd circulator of the lesser world."

During the rest of our interview, Harvey referred to many of the more noticeable episodes of his life,—the favours of royalty; his physicianship at St. Bartholomew's; his travels in Scotland with King Charles; how, by royal order, he came to dissect the body of Old Parr; his travels on the Continent with Dr. Ent, and so on; but these I must deal with later.

Dr. Baldwin Hamey, one of his most intimate friends, has recorded in manuscript the death of Harvey in this quaint style:—"*Gulielmi Har-*

*vaei fortunatissimi Anatomici, desiit sanguis moveri, tertio Idus Junii, 57. Cujus alio qui perennem motum, in omnibus verissimè asserverat.*" And more strangely still, he adds :—"*Sepultus* 26 *Junii*, 1657, *quo die inauguratus est Cromwellus.*"

*Inauguratus* is barely a correct expression to designate the installation of the Lord Protector who has ruled these realms since the death of the martyr King. Some little time after Harvey's funeral I rode over to the village of Chelsea, where Hamey had taken a modest-looking dwelling, there, he tells me, to end his days. Our conversation naturally turned upon Harvey's will, and especially upon that clause which provides—that "Once every year some one shall make an oration publicly in the College, wherein shall be a commemoration of all the benefactors of the said College by name, and what in particular they have done for the benefit of the said College." Harvey expressed the belief that the oration will, in years to come, prove a grand stimulus to the searchers after the great, but as yet hidden, secrets of nature. "What," he asked me, "could be a nobler exhortation to man than that which bids him imitate the great benefactors who have gone before him? If each does this, what can hinder the College from becoming, what I know it will ultimately become, the fountain of knowledge?"

Dr. Hamey, though perhaps not a brilliant man, is one of the most benevolent men of the day. He regards experiment as the basis for the acquisition of all new scientific truths, and here he is heartily at one with his friend whom he loves to call the "great doctor."

When, in 1654, Dr. Prujean resigned the presidency of the College of Physicians, Harvey was elected to succeed him, but he felt that the sand had almost run through the hour-glass, and that, sincerely attached as he was to the institution, it was useless at his age to take upon himself the burden of a heavy responsibility. Thus it was that he met the members, and, while thanking them for the proposed honour, declined the office. Two years later he surrendered to the College a portion of his estate in perpetuity for their use, the object of the gift being the institution of an annual gathering, at which an oration in Latin should be made, to commemorate the benefactors of the College, to afford an honorarium for the orator and a provision for the keeper to the library and museum which he had so generously given.

